# Daratumumab for PRCA after HCT: study and practical considerations from the EBMT Transplant Complications Working Party

**DOI:** 10.1038/s41408-025-01315-8

**Published:** 2025-06-04

**Authors:** Nico Gagelmann, Moniek de Witte, Christophe Peczynski, William Boreland, Annoek E. C. Broers, Edgar Jost, Alexander Kulagin, Albert Esquirol, Simona Sica, Jürgen Kuball, Gerardo Errico, Wolfgang Bethge, Johan Maertens, Friedrich Stölzel, Edouard Forcade, Matthew Collin, Matteo Parma, Goda Choi, Nicolaus Kröger, Maria Chiara Di Chio, Maria Chiara Finazzi, Lucia López Corral, Jose Rifón, Alberto Mussetti, Adrian Bloor, Marco Ladetto, Hélène Schoemans, Olaf Penack, Ivan Moiseev, Zinaida Peric

**Affiliations:** 1 University Medical Center Hamburg-Eppendorf, Hamburg, Germany; 2 Department of Hematology, University Medical Center Utrecht, Utrecht, The Netherlands; 3 EBMT Paris Study Unit, INSERM UMR-S 938, Sorbonne University, Hôpital Saint Antoine, Paris, France; 4 Erasmus MC Cancer Institute, Rotterdam, Netherlands; 5 University Hospital Aachen, Aachen, Germany; 6 RM Gorbacheva Research Institute, Pavlov University, St. Petersburg, Russian Federation; 7 Hospital Santa Creu i Sant Pau, Barcelona, Spain; 8 Universita Cattolica S. Cuore, Rome, Italy; 9 Nottingham City Hospital, Nottingham, UK; 10 Universitaet Tuebingen, Tuebingen, Germany; 11 University Hospital Gasthuisberg, Leuven, Belgium; 12 University Medical Center Schleswig-Holstein, Campus Kiel, Kiel, Germany; 13 CHU Bordeaux, Hopital Haut-Leveque, Pessac, France; 14 RVI Newcastle, Newcastle, UK; 15 Ospedale San Gerardo, Monza, Italy; 16 University Medical Center Groningen, Groningen, Netherlands; 17 University of Milano, Milano, Italy; 18 Secretary and Italian National BMT Registry - GITMO, Bergamo, Italy; 19 Hospital Clínico, Salamanca, Spain; 20 Clínica Universitaria de Navarra, Pamplona, Spain; 21 Institut Catalá d’Oncologia, Hospital Duran i Reynals, Barcelona, Spain; 22 Christie Hospital Manchester, Manchester, UK; 23 H SS. Antonio e Biagio, Alessandria, Italy; 24 Charité-CVK, University Medicine Berlin, Berlin, Germany; 25 University Hospital Centre Rijeka and School of Medicine, University of Rijeka, Rijeka, Croatia

**Keywords:** Anaemia, Drug development

## Abstract

Pure red cell aplasia (PRCA) is a relevant complication after ABO-mismatched allogeneic hematopoietic cell transplantation (HCT). No standard treatment exists, and practice is heterogenous. In this study, we took advantage of an international collaboration to describe characteristics and outcomes of patients receiving daratumumab for PRCA following first allogeneic HCT. We identified 45 patients meeting these criteria (median patient age, 56 years). The median time from HCT to PRCA was 55 days (IQR, 36–116) and all patients were transfusion-dependent at time of daratumumab start. Daratumumab was first-line treatment in 16 patients (36%), most patients (67%) received daratumumab intravenously, and median time from PRCA diagnosis and daratumumab start was 88 days (IQR, 59–219). Incidence of transfusion independence was 69% (95% confidence interval [CI], 52–80%) at 6 months and 80% (95% CI, 62–90%) at 12 months. Incidences of hemoglobin and reticulocyte recoveries were respectively 56 and 78% at 6 months and 65 and 83% at 12 months. Survival at 12 months was 81%, and of 8 deaths, 7 were GVHD- or infection-related. One death was associated with hemolytic anemia. This is the first international and largest study on the use of daratumumab for PRCA after allogeneic HCT, showing high response rates superior to that reported for other treatments. Seven incidents of severe adverse events (mostly infections) underscore the need for close monitoring, proactive management, and comparative studies to determine the role for daratumumab for PRCA. Last, based on these data and a comprehensive literature review, we provide practical consideration for modern PRCA treatment.

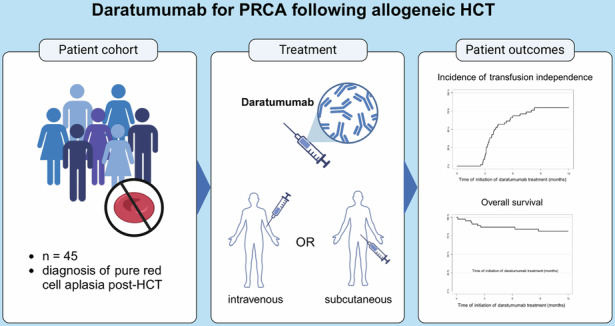

## Introduction

Pure red cell aplasia (PRCA) is a rare but severe complication that can occur following ABO-mismatched allogeneic hematopoietic cell transplantation (HCT) [1]. It is characterized by a selective failure of erythroid progenitors, leading to severe anemia in affected patients [2]. PRCA post-HCT poses significant clinical challenges, often resulting in prolonged dependence on blood transfusions and a reduced quality of life. Despite various therapeutic strategies, including immunosuppressive therapy and donor lymphocyte infusions [3, 4], treatment responses have been inconsistent, highlighting the need for innovative and targeted interventions [5].

Daratumumab, a human anti-CD38 monoclonal antibody, has emerged as a promising therapeutic option in hematologic disorders due to its potent immunomodulatory effects [6]. Initially approved for multiple myeloma, daratumumab has demonstrated the ability to target CD38-expressing plasma cells and immune regulatory cells, leading to the depletion of pathogenic cell populations. Growing evidence, mainly consisting of single cases or small cohorts, suggests that daratumumab may offer clinical benefit in PRCA cases that are refractory to conventional therapies, given its mechanism of targeting and depleting CD38-positive cells that may contribute to the pathogenesis of PRCA [7–12].

This study aimed to explore the characteristics, efficacy, and safety of daratumumab in the management of PRCA following allogeneic HCT, using data from the first and largest international cohort of patients receiving daratumumab in this setting. Finally, we performed a comprehensive literature review to identify all current evidence and positioned our results within the spectrum of currently published literature to give practical considerations concerning the role of daratumumab as novel treatment option for PRCA following allogeneic HCT.

## Methods

### Data collection

The data collection process involved several key steps to ensure the integrity, accuracy, and completeness of the dataset. This study used registry data from the European Society for Blood and Marrow Transplantation (EBMT), serving as a comprehensive repository for collecting and analyzing patient, disease, and transplant-related data across Europe and other collaborating regions. First, we utilized the EBMT network to identify centers that treated patients with daratumumab for post-allogeneic HCT PRCA. Next, we gathered relevant clinical and demographic as well as treatment-specific information on patients diagnosed with PRCA following allogeneic HCT and treated with daratumumab in a real-world setting.

The study adhered to the ethical guidelines established by the EBMT and local regulatory bodies and is in accordance with the Declaration of Helsinki. Informed consent had been obtained from all patients as part of their registration in the EBMT database, in accordance with the organization’s ethical standards.

### Patient selection and inclusion criteria

An invitation was sent to all of EBMT member centres regularly performing adult allogeneic HCTs in order for them to identify eligible patients, as data on PRCA presence was not routinely collected in the registry. The study has been approved by the local ethics committees and patients gave their consent in accordance with EBMT standard practice. This study is in accordance with the Declaration of Helsinki.

The inclusion criteria comprised adult patients who had undergone first allogeneic HCT and were subsequently diagnosed with PRCA, as defined by standard clinical and laboratory criteria [2]. The selection also required patients to have received daratumumab as part of their therapeutic regimen to address PRCA. Treatment with daratumumab in any line of treatment was accepted. Patients with incomplete records or those with other causes of anemia unrelated to PRCA were excluded from the analysis.

The following variables were collected from the 22 EBMT member centres, who accepted to participate, for each of their eligible patients:

- Demographic information: age (patient and donor), gender (patient and donor), ABO mismatch, rhesus factor (patient and donor) and underlying disease for which HCT was performed.
- Transplant-related data: pre-existing comorbidities, conditioning regimen, disease status at HCT, donor type, cell source, performance status at HCT, CMV status (patient and donor), GvHD prophylaxis, previous autologous transplantation, ex-vivo T-cell depletion and history of graft-versus-host disease (GVHD).
- Therapeutic interventions: previous treatments administered for PRCA, schedule and number of infusions of daratumumab, dosage and route of administration, and concurrent therapies.
- Clinical outcomes: hemoglobin levels and reticulocyte counts pre- and post-treatment, transfusion dependency, adverse events, and patient survival rates.

### Study endpoints and statistical analysis

First, we aimed to describe the cohort of patients receiving daratumumab for PRCA, to understand current practices and heterogeneity. Next, we evaluated the outcomes after receiving daratumumab, with particular focus on the main clinical endpoint of transfusion independence (defined as 12 weeks without the need for transfusions). Further endpoints were hemoglobin recovery, reticulocyte recovery, overall survival (OS), and causes of death. OS was defined as time from date of first infusion of daratumumab to latest follow-up or death from any cause.

Probabilities of OS were calculated using the Kaplan–Meier method. Cumulative incidences were used to estimate the endpoints of transfusion independence, hemoglobin recovery and reticulocyte recovery to accommodate for competing risks. Follow-up values were estimated using the reverse Kaplan–Meier method. Statistical analyses were done with R statistical software version 4.1.2.

### Practical considerations

Practical considerations were identified and developed through a consensus process involving a panel of experts with clinical experience in HCT and use of daratumumab for PRCA. Discussions were informed by clinical experience, study and case data, prevailing practice patterns, and interpretation of available but limited data, recognizing that high-quality evidence was lacking in several key areas. The panel acknowledged current challenges and identified areas of unmet clinical need. Through iterative process, position statements and practical considerations were formulated to guide clinical management and provoke future research. Considerations reflected collective expert opinion and professional judgment rather than formal evidence grading.

## Results

### Patient, donor, and transplant characteristics

This study included a total of 45 patients who received daratumumab for PRCA after allogeneic HCT (Table 1). Most of the patients were transplanted between 2020 and 2022, and the most frequent indication for allogeneic HCT was acute leukemia (44%) and myelodysplastic syndrome or myeloproliferative neoplasm (27%). Five patients (11%) received a previous autologous transplantation. Most patients (91%) received allogeneic HCT using peripheral blood. Donor types were as follows: matched sibling (18%), matched unrelated (10/10) (53%), mismatched unrelated (<10/10) (22%), and haploidentical (7%). Most patients (93%) did not receive ex vivo T-cell depletion during allogeneic HCT.

**Table 1:** Patient, donor, and transplant characteristics.

Characteristic	Total cohort
	(*n* = 45)
Patient age in years, median (range)	56.2 (25.8–75.8)
Donor age in years, median (range)	33.2 (16.4–76.3)
Patient sex	
Female	35 (77.8%)
Male	10 (22.2%)
Patient blood group	
O	39 (86.7%)
A	4 (8.9%)
B	2 (4.4%)
Donor blood group	
A	27 (61.4%)
B	11 (25%)
AB	4 (9.1%)
O	2 (4.5%)
Unknown	1
Patient Rhesus	
Present	37 (84)
Absent	7 (16)
Unknown	1
Donor Rhesus	
Present	37 (82)
Absent	8 (18)
Karnofsky score	
90–100%	31 (74)
<90	11 (26)
Unknown	3
HCT-CI	
0	17 (40.5%)
1–2	12 (28.6%)
>2	13 (31%)
Unknown	3
Transplant indication	
Acute leukemia	20 (44.4%)
MDS or MPN	12 (26.7%)
Chronic leukemia	4 (8.9%)
Bone marrow failure	4 (8.9%)
Lymphoma	3 (6.7%)
Autoimmune disease	1 (2.2%)
Other	1 (2.2%)
Disease status at transplant	
Complete remission	22 (50)
Stable disease (no change, no response)	5 (11)
Never treated	4 (9)
Relapse or progression	5 (11)
Other	8 (19)
Unknown	1
Transplant year, median (IQR)	2021 (2020–2022)
Donor type	
Matched unrelated (10/10)	24 (53.3%)
Matched sibling	8 (17.8%)
Mismatched unrelated (<10/10)	10 (22.3%)
Haploidentical	3 (6.7%)
Graft source	
Peripheral blood	41 (91.1%)
Bone marrow	4 (8.9%)
GVHD prophylaxis	
ATG-based	10 (22)
PTCY-based	17 (38)
Other	18 (40)
Ex vivo T-cell depletion	
No	42 (93.3%)
Yes	3 (6.7%)
Conditioning intensity	
Myeloablative	26 (57.8%)
Reduced	19 (42.2%)

The median patient age was 56 years, and median donor age was 33 years. The most frequent patient blood group was O (87%), and the most frequent donor blood group was A (61%). Most patients and donors were male (78 and 84%, respectively).

Intensity of pre-allogeneic HCT conditioning was myeloablative in 58% and reduced in 42% of patients. Total-body irradiation was used as part of the conditioning in 18%. The most frequently used conditioning regimen was busulfan-fludarabine (42%) or busulfan-fludarabine-thiotepa (20%). The most frequent GVHD prophylaxis was cyclosporine A and mycophenolate mofetil (16%).

### Characteristics of daratumumab for PRCA

The median onset of PRCA diagnosis following HCT was 55 days (interquartile range [IQR], 36–116 days). All patients were transfusion-dependent upon daratumumab start and needed at least weekly transfusions (Table 2). Daratumumab was used as first-line treatment in 16 patients (36%), while the remaining 29 patients (64%) received other treatments prior to daratumumab start. Of those, 26 (90%) received rituximab, 6 (21%) received cyclosporine A, and 10 (34.5%) received steroids for PRCA.

**Table 2:** PRCA and daratumumab characteristics.

Characteristic	Total cohort
	(*n* = 45)
Time between transplant and PRCA in days, median (range)	55 (36–116)
Treatments for PRCA before daratumumab	29 (64.4%)
Rituximab	26 (89.7%)
Steroids	10 (34.5%)
Cyclosporine A	6 (20.7%)
Other^a^	9 (31.0%)
Patient transfusion dependent at daratumumab start	45 (100%)
Total number of RBC transfusions 8 weeks before daratumumab, median (IQR)	8 (6–10.5)
Hemoglobin level at daratumumab start in g/dL, median (range)	7.4 (2.4–9.5)
Ferritin level at daratumumab start in µg/l, median (range)	2707 (1025–7883)
Unknown	15
Time between PRCA and daratumumab start in days, median (range)	88 (59–219)
Route of administration of daratumumab	
Intravenous	30 (66.7%)
Subcutaneous	15 (33.3%)
Number of daratumumab infusions	
1	4 (9.3%)
2	14 (32.6%)
3	3 (7%)
4	5 (11.6%)
5	1 (2.3%)
6	12 (27.9%)
8	4 (9.3%)
Unknown	2
Schedule of infusions of daratumumab	
1× per week	28 (62.2%)
1× per 2 weeks	5 (11.1%)
1× per month	3 (6.7%)
Only 1 infusion	4 (8.9%)
Other	5 (11.1%)
Duration of daratumumab treatment in days, median (IQR)	21 (10–39)

^a^Bortezomib in 3 patients, plasma exchange in 4 patients, Darbepoietin in 1 patient, Erythropoietin in 1 patient.

The median time between PRCA diagnosis and daratumumab treatment was 88 days (IQR, 59–219 days), and the median duration of daratumumab treatment was 21 days (IQR, 10–39 days). Daratumumab was given intravenously in 30 patients (67%) or subcutaneously in 15 patients (33%). Infusion schedules were as follows: once per week in 28 patients (62%); once every two weeks in five patients (11%); once every four weeks in three patients (7%); and only given once in four patients (9%). Five patients received other schedules: one patient received infusions at day 1 and 10; one patient received infusions at day 1, 8, and 22 given for two cycles; one patient received one cycle of infusions at day 1, 8, and 22; one patient received an infusion once every week or two weeks for 18 infusions; and one patient received two weekly infusions, two every two weeks, and two monthly. The most frequent dosing of daratumumab was 16 mg/kg bodyweight in 37 patients (82%). Four patients received lower doses (two patients with 8 mg/kg, one patient with 12 mg/kg, and one patient with 13 mg/kg, respectively). The remaining four patients received higher doses.

### Efficacy of daratumumab for PRCA

The median follow-up after daratumumab treatment was 17.4 months (95% confidence interval [CI], 11.1–27.3 months) for the total cohort of 45 patients. Incidence of transfusion independence, defined as 12 weeks without transfusions, was 69% (95% CI, 52–80%) at 6 months and 80% (95% CI, 62–90%) at 12 months after daratumumab start. Only one patient had documented PRCA relapse at 39 days after start of daratumumab treatment. Incidence of hemoglobin recovery at 12 months was 65% (95% CI, 48–77%). Incidence of reticulocyte recovery at 12 months was 83% (95% CI, 65–93%).

**Fig. 1:**
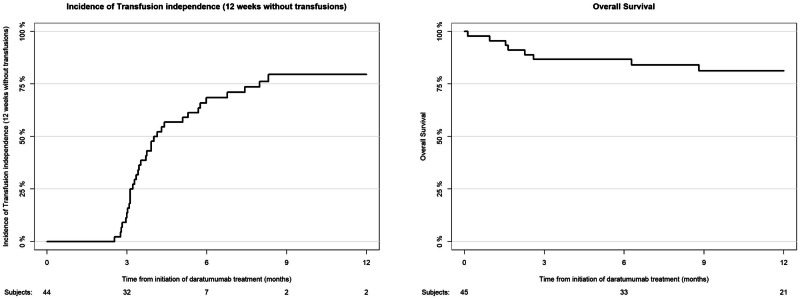
Cumulative incidence of transfusion dependence and overall survival of the total cohort. In 45 patients, median follow-up was 17.4 months. Transfusion independence was 69% at 6 months and 80% at 12 months, with one PRCA relapse. At 12 months, hemoglobin and reticulocyte recovery were 65 and 83%, respectively. Overall survival was 87% at 6 months and 81% at 12 months, with 8 deaths mostly related to HCT complications.

The overall survival was 87% (95% CI, 77–97%) at 6 months and 81% (95% CI, 70–94%) at 12 months after start of daratumumab treatment (Fig. 1). In total, eight deaths occurred, of which one was caused by a relapse of the original disease and the remaining seven were allogeneic HCT-related. Only one patient died from complications by hemolytic anemia, while the remaining patients died from GVHD and/or co-occurring infections with multiorgan failure.

### Safety of daratumumab for PRCA

Serious adverse events associated with daratumumab were observed in seven patients. Among the most frequently reported severe adverse events, classified as Grade 3 or higher according to the Common Terminology Criteria for Adverse Events (CTCAE), were various types of infections. These included SARS-CoV-2 pneumonia, pneumocystis pneumonia, cytomegalovirus (CMV) reactivation, and an unspecified infection, each affecting one patient.

Additionally, one patient experienced severe diarrhea, which was identified as an adverse event directly linked to daratumumab administration. Another case involved an anaphylactic shock, a potentially life-threatening allergic reaction, while yet another patient suffered from severe respiratory failure. These incidents highlight the range of serious complications that can occur in association with daratumumab treatment, underscoring the need for close monitoring and proactive management of such adverse effects.

## Discussion

This is the first multicenter international cohort study on the use of daratumumab for PRCA following allogeneic HCT, providing valuable insights into the characteristics and use of daratumumab for managing PRCA. Our cohort of 45 patients illustrates the potential clinical efficacy of daratumumab in reducing transfusion dependence, resulting in promising overall survival for this challenging post-transplant complication.

A notable finding is the high rate of transfusion independence achieved among patients following daratumumab treatment. With a 69% incidence of transfusion independence at 6 months and 80% at 12 months post-daratumumab. This treatment has demonstrated robust clinical efficacy in promoting erythroid recovery in patients with PRCA after allogeneic HCT. This is particularly promising, given that all patients were transfusion-dependent before initiating daratumumab, with some requiring daily transfusions. Most studies and case reports reported fast disappearance of iso-hemaglutinnin in almost all patients, with few cases of persistence and PRCA relapse [7, 8, 12–14]. In comparison to currently published results of daratumumab for PRCA, our incidence of transfusion-independence appeared to be lower (80% [at one-year after start of daratumumab] versus 93%). However, follow-up is still limited and non-responsive cases are often not reported in the literature, leading to publication bias and potentially overestimating treatment efficacy. Furthermore, most case reports had short follow-up, and actual clinical benefit for patients may be better measured by transfusion-free survival with long-term follow-up, since transfusion-dependency is associated with reduced quality of life and significant morbidity [15]. Recent multicenter studies showed evidence of PRCA relapse or persistence in some cases [16]. In line with these reports, our study provides first strong evidence for low relapse rates, but even larger cohorts are needed to better understand mechanisms of non-response and relapse.

Overall, the outcomes related to hemoglobin recovery and overall survival further support the potential of daratumumab as an effective therapeutic option for PRCA. Hemoglobin recovery, achieved in 65% of patients at 12 months, along with an overall survival of 81% at 12 months, suggests that daratumumab not only facilitates transfusion independence but also enhances hematologic recovery and survival in these high-risk patients. Importantly, only one case of PRCA relapse was documented, and only one patient experienced mortality due to hemolytic anemia, indicating durable responses and manageable safety outcomes. This underscores the complexity of managing post-allogeneic HCT complications and the need for ongoing supportive care and monitoring in this patient population.

In terms of infections, as CD38 is expressed at the surface of activated CD4+T cells, this study cannot rule out that daratumumab might have played a role in predisposing patients to infections, such as pneumocystis or viral infections [16, 17].

The relatively rapid median onset of PRCA following allogeneic HCT (55 days) underscores the need for early and effective intervention, which daratumumab appears to provide for the majority of patients. Daratumumab can induce complete response and transfusion-independence even after a single application. Furthermore, the alternative of subcutaneous application may offer patients and physicians alike to act timely where PRCA treatment is indicated.

To the best of our knowledge, this is the largest report of patients receiving daratumumab even as first-line treatment for PRCA, with promising responses compared with historical cohorts. A systematic literature review was conducted to evaluate the current role of daratumumab for PRCA. Relevant studies were identified through a comprehensive search of PubMed, with no language or publication date restrictions to ensure an inclusive dataset. Search terms included “daratumumab,” “pure red cell aplasia,” “PRCA,” “CD38 monoclonal antibody,” and “transplantation,” combined with Boolean operators. A qualitative synthesis of findings was conducted due to heterogeneity in study design and outcome reporting across studies, identifying 15 articles describing a total of 46 patients (Table 3) [7–9, 11–13, 16, 18–26]. Only 5 studies reported more than one patient. Most frequent administration route in case reports was intravenous, while the two largest studies to date included more patients with subcutaneous daratumumab. Synthesized response rate over all reports (except the present one) with >1 patient was 87%, and most responses occurred early after a median of two doses. In contrast, all case reports showed response in each patient.

**Table 3:** Current reports on daratumumab for PRCA.

Publication	*N*	Diagnosis	Prior treatments	Dosing	Doses	Treatment start from HCT in days	Response
Weverling et al.	14	AML, MDS, MPN, SCD, lymphoma	rituximab (43%), DLI (29%), bortezomib (7%)	1800 mg SC	1–6	60–2989	93% (defined by achievement of reticulocytosis), no relapse
Longval et al.	11	AML, MDS, MPN, SAA, DADA2	rituximab, EPO, thrombopoietin receptor agonist	7 with IV and 4 with SC	Median of 3	Median of 255	91% (defined by transfusion independence)
Giammarco et al.	6	Hematological diseases	rituximab, EPO, thrombopoietin receptor agonist	1800 mg SC	2	NA	83% (defined by transfusion independence)
Frioni et al.	3	NR	EPO, plasma exchange, rituximab	1800 mg SC	2–8	100–180	2 responses, 1 with no response after 8 doses
Desai et al.	3	AA, AML	Tapering of IS, IVIG, rituximab, steroids, plasma exchange, bortezomib	2 IV and 1 SC	4–6	180–730	100%
Chapuy et al.	1	MDS	tapering of IS, steroids, rituximab, darbepoetin	16 mg/kg IV	6	390	After first dose
Bathini et al.	1	SAA	Tapering of IS, Steroids, rituximab, bortezomib	16 mg/kg IV	4	411	After first dose
Rautenberg et al.	1	AML	Tapering of IS, rituximab	16 mg/kg IV	2	206	After second dose
Salas et al.	1	AA	Tapering of IS, rituximab, steroids, plasma exchange bortezomib	16 mg/kg IV	6	700	After 6^th^ dose
Henig et al.	1	Chronic neutropenia	Tapering of IS, rituximab, bortezomib	16 mg/kg IV	5	320	After 4^th^ dose
Yates et al.	1	DOCK8 deficiency	Tapering of IS	16 mg/kg IV	3	397	After third dose
Martino et al.	2	AML, MDS	NR	16 mg/kg IV	2, 3	205, 270	After first and second dose
Asawapanumas et al.	1	AML	Tapering IS	16 mg/kg IV	1	146	After first dose
Wu et al.	1	MDS	Tapering IS, rituximab	16 mg/kg IV	3	209	After second dose
Deng et al.	1	AML	Tapering IS, plasma exchange, interferon, MSC	16 mg/kg IV	5	2701	After second dose
Dovern et al.	1	SCD	None	1800 mg SC	4	60	After second dose

*IV* intravenous, *SC* subcutaneous, *IS* immunosuppression, *MSC* mesenchymal stem cells, *IVIG* intravenous immunoglobulins, *MDS* myelodysplastic neoplasm, *AML* acute myeloid leukemia, *SCD* sickle cell disease, *AA* aplastic anemia, *MPN* myeloproliferative neoplasm, *NR* not reported.

Daratumumab’s mechanism of targeting CD38 on immune cells, particularly on plasma cells that may contribute to PRCA pathogenesis, likely plays a key role in its therapeutic effect. This immunomodulatory function is especially relevant in our cohort, where 93% of patients received in vivo T-cell depletion, a factor often associated with increased risk of PRCA due to reduced immune regulation [27]. The effective use of daratumumab in patients already heavily pretreated for PRCA, including 64% who had received alternative therapies like rituximab, cyclosporine A, and steroids, also highlights its potential role as a valuable second-line or salvage therapy when conventional treatments fail to yield desired outcomes.

However, the high number of patients that died of GvHD suggests that while daratumumab effectively targets PRCA, it does not mitigate all allogeneic HCT-related risks, particularly those associated with immune reconstitution and GVHD. This finding highlights the need for further studies to investigate combination and frontline strategies that may complement daratumumab’s efficacy while also addressing GVHD and infection risks.

PRCA following allogeneic HCT exerts a profound and multifaceted impact on patient quality of life, compounding the already demanding post-transplant recovery period. The near-universal transfusion dependence seen in this cohort reflects a daily reality of physical fatigue, reduced functional capacity, and frequent hospital visits that disrupt personal and social routines. Patients often experience emotional distress from prolonged uncertainty and delayed engraftment, especially when PRCA proves refractory to initial immunosuppressive therapies. The median 88-day delay before initiating daratumumab underscores a period of clinical vulnerability that severely diminish overall well-being. Additionally, the psychological toll of needing continuous transfusions and facing the risk of iron overload or alloimmunization can erode a patient’s sense of autonomy and long-term hope. While daratumumab ultimately restored transfusion independence in most cases, the interim period of symptomatic anemia, hospital dependence, and anxiety about transplant failure defines PRCA as a condition with heavy physical and psychosocial burdens that extend well beyond its hematologic profile.

While the present study focused on the treatment of PRCA after HCT, prevention of PRCA, particularly in the setting of major ABO mismatch, remains a clinical challenge, but several strategies have been proposed to mitigate its risk. For instance, decrease of isoagglutinin titers by in vivo immunoadsorption before allogeneic HCT does not only lack severe complication but also leads to a reduction in demand of RBC transfusion after engraftment and may reduce the incidence of PRCA in these patients [28]. Furthermore, use of a standardized pretransplant isoagglutinin reduction strategy including donor-type secretor plasma infusions is both safe and efficient in preventing progenitor cell infusion-associated hemolysis and is associated with low rates of post-transplant PRCA [29]. Post-transplant strategies, such as early tapering of immunosuppression or the use of rituximab in high-risk mismatched transplants, have also been proposed to facilitate donor erythroid engraftment. Furthermore, graft manipulation techniques like CD34+ selection or T-cell depletion may impact the risk, though data remain inconclusive. Despite these efforts, no standardized preventive protocol exists, and approaches are typically tailored based on institutional experience and individual patient risk factors.

We acknowledge limitations of the present study. We did not have systematic data on antibody titers before and after daratumumab treatment, as well as detailed data on dosing and timing of treatment lines other than daratumumab (such as cyclosporine A, rituximab, steroids). The application schedule and dosing of daratumumab varied significantly, reflecting the individualized approach often needed in complex allogeneic HCT patients. The most common dosing regimen, 16 mg/kg, administered weekly, aligns with standard daratumumab dosing in other hematologic indications. Another option in addition to daratumumab could be the other anti-CD38 antibody isatuximab that showed activity in a case report [30]. To address its potential, a randomized trial is currently recruiting participants to investigate the efficacy of isatuximab after a “watch and wait” phase in ABO-mismatched PRCA (NCT05559827). Last, recent reports descriped successful treatment of PRCA in major ABO-mismatched allogeneic HCT with single agent BTK inhibition such as with ibrutinib [31]. In our cohort, none of the patients received BTK inhibition and future studies should focus on novel treatment sequences in non-responders as well as identification of subgroups that may benefit from individual treatment approaches.

In conclusion, this study demonstrates that daratumumab may be a promising treatment option for PRCA following allogeneic HCT, with the ability to reduce transfusion dependency, support hemoglobin recovery, and improve survival. Future research should focus on optimizing and harmonizing dosing and administration schedules, as well as exploring combination therapies to further reduce post-allogeneic HCT complications and enhance patient outcomes in this vulnerable population.

Earlier daratumumab use, standard dosing, and possibly fewer prior therapies seem associated with better outcomes, while delayed treatment and severe infections, and yet unknown complex transplant variables may worsen prognosis and influence treatment adherence. Based on this, we provided expert positions suggesting the early use and first-line treatment, especially for patients with severe anemia, rapid clinical decline, or contraindications to standard immunosuppressive therapies (Table 4). By adhering to these considerations, clinicians can maximize the therapeutic benefits of daratumumab while minimizing risks, develop studies, ultimately improving outcomes and quality of life for patients with PRCA following allo-HCT.

**Table 4:** Practical considerations.

Patient selection:
◦ Use daratumumab early in transfusion-dependent PRCA, especially within 2–3 months post-HCT.
◦ Consider first-line use in patients with severe anemia, rapid clinical decline, or contraindications to standard immunosuppressive therapies.
• Administration and dosing:
◦ Preferred regimens:
▪ IV infusion: 16 mg/kg weekly for 4–6 doses.
▪ SC injection: 1800 mg weekly, offering similar efficacy with greater convenience.
◦ Reassess response after 2–4 doses; consider extending to 8 doses if needed.
• Monitoring and supportive care:
◦ Employ full infection prophylaxis and monitor for CMV reactivation and other infections.
◦ Use premedications to prevent infusion reactions; monitor closely during initial doses.
◦ Coordinate care with transplant and infectious disease teams to manage GVHD and infection risks.
• Follow-up and long-term care:
◦ Monitor hemoglobin and reticulocyte counts biweekly for 3 months, then quarterly for 1 year.
◦ Be vigilant for PRCA relapse; re-treatment with daratumumab may be considered if necessary.
• Therapeutic positioning:
◦ Prioritize daratumumab over rituximab in settings requiring rapid hematologic response.
◦ Consider combination approaches or alternative anti-CD38 agents for refractory or relapsed PRCA.

## Data Availability

Data will be made available upon reasonable request to the corresponding author.

## References

[1] Marco-Ayala J, Gomez-Segui I, Sanz G, Solves P. Pure red cell aplasia after major or bidirectional ABO incompatible hematopoietic stem cell transplantation: to treat or not to treat, that is the question. Bone Marrow Transpl. 2021;56:769–78. 10.1038/s41409-020-01124-6. 10.1038/s41409-020-01124-6 33188257

[2] Means RT Jr. Pure red cell aplasia. Hematol Am Soc Hematol Educ Program. 2016;2016:51–56. 10.1182/asheducation-2016.1.51. 10.1182/asheducation-2016.1.51 PMC6142432 27913462

[3] Santamaria A, Sureda A, Martino R, Domingo-Albos A, Muniz-Diaz E, Brunet S. Successful treatment of pure red cell aplasia after major ABO-incompatible T cell-depleted bone marrow transplantation with erythropoietin. Bone Marrow Transpl. 1997;20:1105–7. 10.1038/sj.bmt.1701012. 10.1038/sj.bmt.1701012 9466287

[4] Sora F, De Matteis S, Piccirillo N, Chiusolo P, Laurenti L, Putzulu R, et al. Rituximab for pure red cell aplasia after ABO-mismatched allogeneic peripheral blood progenitor cell transplantation. Transfusion. 2005;45:643–5. 10.1111/j.0041-1132.2005.00445.x. 15819690 10.1111/j.0041-1132.2005.00445.x

[5] Means RT Jr. Pure red cell aplasia: the second hundred years. Am J Med Sci. 2023;366:160–6. 10.1016/j.amjms.2023.06.009. 37327996 10.1016/j.amjms.2023.06.009

[6] de Weers M, Tai YT, van der Veer MS, Bakker JM, Vink T, Jacobs DC, et al. Daratumumab, a novel therapeutic human CD38 monoclonal antibody, induces killing of multiple myeloma and other hematological tumors. J Immunol. 2011;186:1840–8. 10.4049/jimmunol.1003032. 21187443 10.4049/jimmunol.1003032

[7] Chapuy CI, Kaufman RM, Alyea EP, Connors JM. Daratumumab for delayed red-cell engraftment after allogeneic transplantation. N Engl J Med. 2018;379:1846–50. 30403942 10.1056/NEJMoa1807438

[8] Rautenberg C, Kaivers J, Germing U, Haas R, Ackerstaff S, Hoffmann T, et al. Daratumumab for treatment of pure red cell aplasia after allogeneic stem cell transplantation. Bone Marrow Transpl. 2020;55:1191–3. 10.1038/s41409-019-0664-4. 10.1038/s41409-019-0664-4 31481799

[9] Asawapanumas T, Chanswangphuwana C, Watanaboonyongcharoen P, Rojnuckarin P, Bunworasate U. Daratumumab as a frontline immunosuppression for pure red cell aplasia after major ABO-mismatched allogeneic hematopoietic stem cell transplantation. Leuk Res Rep. 2022;17:100314. 10.1016/j.lrr.2022.100314. 35509968 10.1016/j.lrr.2022.100314 PMC9059067

[10] Gangat N, Bleeker J, Lynch D, Olteanu H, Letendre L, Tefferi A. Daratumumab for treatment-refractory acquired idiopathic pure red cell aplasia. Haematologica. 2022;107:2523–6. 10.3324/haematol.2022.281398. 35678030 10.3324/haematol.2022.281398 PMC9521224

[11] Martino R, Garcia-Cadenas I, Esquirol A. Daratumumab may be the most effective treatment for post-engraftment pure red cell aplasia due to persistent anti-donor isohemagglutinins after major ABO-mismatched allogeneic transplantation. Bone Marrow Transpl. 2022;57:282–5. 10.1038/s41409-021-01507-3. 10.1038/s41409-021-01507-3 PMC8552208 34711914

[12] Weverling F, Roeven M, Nijssen C, Broers AEC, Dovern E, van Rhenen A, et al. Efficacy and safety of daratumumab in pure red cell aplasia after allogeneic transplantation: Dutch real-world data. Blood Adv. 2024;8:1683–6. 10.1182/bloodadvances.2023011190. 38231018 10.1182/bloodadvances.2023011190 PMC11006807

[13] Bathini S, Holtzman NG, Koka R, Singh Z, Wilding E, Zou Y, et al. Refractory postallogeneic stem cell transplant pure red cell aplasia in remission after treatment with daratumumab. Am J Hematol. 2019;94:E216–E219. 10.1002/ajh.25515. 31120638 10.1002/ajh.25515

[14] Longval T, Galimard JE, Lepretre AC, Suarez F, Amiranoff D, Cazaux M, et al. Treatment for pure red cell aplasia after major ABO-incompatible allogeneic stem cell transplantation: a multicentre study. Br J Haematol. 2021;193:814–26. 10.1111/bjh.17463. 33844842 10.1111/bjh.17463

[15] Melchert M, List AF. Management of RBC-transfusion dependence. Hematol Am Soc Hematol Educ Program. 2007:398–404. 10.1182/asheducation-2007.1.398 10.1182/asheducation-2007.1.398 18024657

[16] Longval T, Lepretre AC, Ravinet A, Fayard A, Forcade E, Coman T, et al. Efficacy and safety of Daratumumab for the treatment of ABO-incompatible pure red cell aplasia after allogenic HSCT: report from SFGM-TC. Bone Marrow Transpl. 2024;59:893–5. 10.1038/s41409-024-02202-9. 10.1038/s41409-024-02202-9 38461290

[17] Li W, Liang L, Liao Q, Li Y, Zhou Y. CD38: an important regulator of T cell function. Biomed Pharmacother. 2022;153:113395 10.1016/j.biopha.2022.113395. 35834988 10.1016/j.biopha.2022.113395

[18] Henig I, Yehudai-Ofir D, Zohar Y, Zuckerman T. Pure red cell aplasia following ABO-mismatched allogeneic hematopoietic stem cell transplantation: resolution with daratumumab treatment. Acta Haematol. 2021;144:683–7. 10.1159/000515257. 33887733 10.1159/000515257

[19] Wu C, Manchen P, Edelman A, Husnain M, Katsanis E, Fuchs D, et al. Refractory pure red blood cell aplasia secondary to major ABO-incompatible allogeneic stem cell transplantation successfully treated with daratumumab. J Hematol. 2023;12:277–82. 10.14740/jh1195. 38188476 10.14740/jh1195 PMC10769644

[20] Dovern E, Biemond BJ, Nur E. Case report: successful treatment with daratumumab for pure red cell aplasia in a patient with mixed lymphoid chimerism after ABO-mismatched stem cell transplant for sickle cell disease. Front Immunol. 2023;14:1212007. 10.3389/fimmu.2023.1212007. 37426651 10.3389/fimmu.2023.1212007 PMC10326381

[21] Deng B, Gao R, Yang B, Lei WB, Xue MF, Wang JS, et al. Seven-years post allogeneic hematopoietic stem cell transplantation pure red cell aplastic anemia cured with daratumumab: a case report and review of literature. World J Clin Cases. 2024;12:5604–12. 10.12998/wjcc.v12.i24.5604. 39188601 10.12998/wjcc.v12.i24.5604 PMC11269989

[22] Yates B, Molloy E, Dulau-Florea A, Braylan R, Hogan L, Hickstein DD, et al. Daratumumab for delayed RBC engraftment following major ABO mismatched haploidentical bone marrow transplantation. Transfusion. 2021;61:1041–6. 10.1111/trf.16281. 33528026 10.1111/trf.16281

[23] Frioni F, Metafuni E, Limongiello MA, Piccirillo N, Massini G, Pellegrino C, et al. Posttransplant autoimmune hemolytic anemia with anti-D specificity successfully treated with daratumumab: a case report. Transfus Med Hemother. 2024;51:355–8. 10.1159/000535927. 39371256 10.1159/000535927 PMC11452148

[24] Salas MQ, Alahmari A, Lipton JH. Successful treatment of refractory red cell aplasia after allogeneic hematopoietic cell transplantation with daratumumab. Eur J Haematol. 2020;104:145–7. 10.1111/ejh.13343. 31693245 10.1111/ejh.13343

[25] Giammarco S, Limongiello MA, Di Marino L, Metafuni E, Teofili L, Chiusolo P, et al. The role of daratumumab in complications post-allogeneic hematopoietic stem cell transplantation: a single-center prospective study on PRCA and AIHA. Bone Marrow Transpl. 2024. 10.1038/s41409-024-02479-w. 10.1038/s41409-024-02479-w 39567767

[26] Desai N, Viswabandya A, Kim DDH, Lipton JH, Mattsson J, Law AD. Daratumumab in the management of red cell aplasia following allogeneic hematopoietic stem cell transplantation. Eur J Haematol. 2025;114:310–4. 10.1111/ejh.14341. 39494777 10.1111/ejh.14341

[27] Gomez-Arteaga A, Scordo M, Tamari R, Ruiz JD, Jakubowski AA, Papadopoulos EB, et al. Use of anti-thymocyte globulin (ATG) for the treatment of pure red cell aplasia and immune-mediated cytopenias after allogeneic hematopoietic cell transplantation: a case series. Bone Marrow Transpl. 2020;55:2326–30. 10.1038/s41409-020-0939-9. 10.1038/s41409-020-0939-9 32424188

[28] Scholl S, Klink A, Mugge LO, Schilling K, Hoffken K, Sayer HG. Safety and impact of donor-type red blood cell transfusion before allogeneic peripheral blood progenitor cell transplantation with major ABO mismatch. Transfusion. 2005;45:1676–83. 10.1111/j.1537-2995.2005.00578.x. 16181220 10.1111/j.1537-2995.2005.00578.x

[29] Curley C, Pillai E, Mudie K, Western R, Hutchins C, Durrant S, et al. Outcomes after major or bidirectional ABO-mismatched allogeneic hematopoietic progenitor cell transplantation after pretransplant isoagglutinin reduction with donor-type secretor plasma with or without plasma exchange. Transfusion. 2012;52:291–7. 10.1111/j.1537-2995.2011.03295.x. 21848968 10.1111/j.1537-2995.2011.03295.x

[30] Nauffal M, Eng S, Lin A, Chan A, Mazzerella K, Giralt S, et al. Isatuximab for delayed red cell engraftment after allogeneic hematopoietic cell transplantation. Case Rep Hematol. 2024;2024:5790011 10.1155/2024/5790011. 39246802 10.1155/2024/5790011 PMC11379505

[31] Arslan S, Ali H, Mei M, Marcucci G, Forman S, Nakamura R, et al. Successful treatment of refractory pure red cell aplasia in major ABO-mismatched allogeneic hematopoietic stem cell transplant with single agent Ibrutinib. Bone Marrow Transpl. 2022;57:830–3. 10.1038/s41409-022-01590-0. 10.1038/s41409-022-01590-0 35194155

